# Wear of Polymer-Infiltrated Ceramic Network Materials against Enamel

**DOI:** 10.3390/ma15072435

**Published:** 2022-03-25

**Authors:** Jumpei Tokunaga, Hiroshi Ikeda, Yuki Nagamatsu, Shuji Awano, Hiroshi Shimizu

**Affiliations:** 1 Division of Biomaterials, Department of Oral Functions, Kyushu Dental University, Fukuoka 803-8580, Japan; r18tokunaga@fa.kyu-dent.ac.jp (J.T.); yuki-naga@kyu-dent.ac.jp (Y.N.); r14shimizu@fa.kyu-dent.ac.jp (H.S.); 2 Division of Clinical Education Development and Research, Department of Oral Functions, Kyushu Dental University, Fukuoka 803-8580, Japan; awa-shu@kyu-dent.ac.jp

**Keywords:** PICN, ceramic, mechanical properties, crown, restorative materials, dental materials

## Abstract

Polymer-infiltrated ceramic network materials (PICNs) have high mechanical compatibility with human enamel. However, the wear properties of PICN against natural human enamel have not yet been clarified. We investigated the in vitro two-body wear behaviors of PICNs and an enamel antagonist. Two PICNs were used: Experimental PICN (EXP) prepared via the infiltration of methacrylate-based resin into the porous silica ceramic network and commercial Vita Enamic (ENA). Two commercial dental ceramics, lithium disilicate glass (LDS) and zirconia (ZIR), were also characterized, and their wear performance was compared to PICNs. The samples were subjected to Vickers hardness tests and two-body wear tests that involve the samples being cyclically impacted by enamel antagonists underwater at 37 °C. The results reveal that the Vickers hardness of EXP (301 ± 36) was closest to that of enamel (317 ± 17). The volumetric wear losses of EXP and ENA were similar to those of LDS but higher than that of zirconia. The volumetric wear loss of the enamel antagonist impacted against EXP was moderate among the examined samples. These results suggest that EXP has wear behavior similar to that of enamel. Therefore, PICNs are mechanically comparable to enamel in terms of hardness and wear and are excellent tooth-restoration materials.

## 1. Introduction

Computer-aided design and computer-aided manufacturing (CAD/CAM) systems for fabricating dental prostheses have improved significantly in recent years [1]. Along with this advancement, dental ceramics for CAD/CAM systems have been developed for use as crown- and bridge-restorative materials [2]. The mechanical properties of contemporary CAD/CAM ceramics used in clinical practice, zirconia (ZIR) and lithium disilicate glass (LDS), have been enhanced to use as all-ceramic crowns [3]. CAD/CAM ceramics have been implemented in practical applications because of their excellent biocompatibility and mechanical properties. However, the mechanical properties of CAD/CAM ceramics are still significantly different from those of human teeth. For instance, the flexural strength of ZIR is the highest among contemporary dental ceramics, enabling its long-term use as crowns and bridges, even as molars where a high gritted force is generated [4]. On the other hand, the surface hardness of ZIR is significantly higher than that of enamel; therefore, ZIR crowns have a risk of abrasion of the opposing teeth [5]. LDS, belonging to the glass-ceramic category, has a natural-looking aesthetic because of its color and transparency due to the glass matrix phase present [6]. LDS has high strength and can be used as an all-ceramic crown without a metal base, unlike conventional porcelain-fused metal crowns. However, similar to ZIR, LDS has a hard surface and can damage the enamel antagonist [5].

Polymer-infiltrated ceramic network materials (PICN) have been investigated for crown restoration due to their high mechanical compatibility with human enamel. PICNs are prepared from pre-sintered porous ceramics immersed in a resin monomer and then polymerized. PICNs have intermediate properties between those of resins and ceramics because of their unique microstructure consisting of a dual-network structure of the ceramic skeleton and polymer phases [7]. The similarities of PICNs to human teeth, including enamel and dentin, have been investigated in terms of mechanical properties such as flexural strength, surface hardness, and elastic modulus [7,8,9,10,11,12]. For instance, Coldea et al. demonstrated that the Vickers hardness and elastic modulus of PICNs are closer to those of natural teeth than those of contemporary dental materials [13]. In recent years, considerable efforts have been made to develop novel PICNs to mimic tooth morphology. Eldafrawy et al. developed a functionally graded PICN whose flexural strength, flexural modulus, and hardness gradually declined to imitate a real tooth structure [14]. Sodeyama developed a 3D-printable PICN with an elastic modulus well-matched to dentin and a Vickers hardness compatible with enamel [15]. Li et al. developed a ZIR-based PICN, which possessed an enamel-like microstructure and mechanical properties [16]. Therefore, from a biomimetic aspect, PICN materials are excellent tooth-restorative materials [17].

Dental crowns and opposing enamel antagonists in the oral cavity gradually wear each other through chewing and bruxism. Therefore, the wear property of crown-restorative materials is an important mechanical property for the long-term clinical success of tooth restoration. For dental CAD/CAM ceramics, numerous in vitro studies have been conducted to examine the wear behaviors of dental-restorative materials under simulated oral environments by comparing each material combination [18,19,20,21,22,23,24,25,26]: Ceramic vs. enamel antagonist, ceramic vs. ceramic antagonist, and enamel vs. enamel antagonist. Many studies have been conducted on the wear behavior of ZIR and LDS. Compared to ceramics research, in vitro studies on the wear of PICNs are limited [27,28,29,30,31,32]. For instance, Zhawi et al. found that monolithic crowns made from PICN exhibited excellent resistance to sliding contact fatigue and wear [33]. Xu et al. reported that commercial PICN exhibited a wear damage mode similar to enamel [28]. For comparative studies of PICN and ceramics, Wille et al. investigated the wear behaviors of PICN, LDS, and ZIR-reinforced LDS against a steatite antagonist [31]. Turker et al. investigated the volumetric wear loss of CAD/CAM materials (PICN, resin composite, ZIR, and ZIR-reinforced LDS) against an enamel antagonist [30]. However, in terms of wear behavior, the mechanical compatibility of PICNs with enamel has not yet been clarified.

This study evaluated the two-body wear of PICNs against an enamel antagonist in vitro. Two PICNs were used, one commercial and an experimental PICN. The commercial PICN (ENA) was Vita Enamic, which has been widely used in both clinical practice and fundamental research for over 10 years. Enamic is the most-popular PICN used in dentistry. Experimental PICN (EXP) was developed for a novel CAD/CAM block that is mechanically compatible with human teeth [11,34]. For comparison, two commercial dental ceramics were also evaluated: LDS and ZIR. The ZIR used in this experiment was tetragonal zirconia polycrystal stabilized with 3 mol% yttria (3Y-TZP), which has superior mechanical properties among dental ceramics. These ceramics are widely used as crown-restorative materials worldwide. The PICNs, ceramics, and enamel antagonists were characterized for Vickers hardness and volumetric wear loss.

## 2. Materials and Methods

### 2.1. Experimental PICN

Experimental PICN (EXP) was prepared according to the process reported in our previous study [34]. Table 1 lists the reagents used to prepare EXP. The starting materials, silica (SiO_2_: 24.0 g), 2-hydroxyethyl methacrylate (HEMA: 7.2 g), triethylene glycol dimethacrylate (TEGDMA: 0.8 g), 2-phenoxyethanol (POE: 1.6 g), 1-propanol (PrOH: 6.4 g), and phenylbis (2,4,6-trimethylbenzoyl) phosphine oxide (BAPO: 0.4 g), were mixed using a planetary centrifugal mixer (ARE-310, THINKY Corp., Tokyo, Japan) operated at 2000 rpm for 6 min followed by 2200 rpm for 1 min in defoaming mode. The obtained slurry was poured into a transparent silicone mold and light-cured using a light irradiator (α-LIGHT II N, J. Morita Corp., Osaka, Japan) for 10 min, resulting in the formation of a precursor block. The block was dried in an oven at 80 °C for seven days. The dried blocks were sintered at 1150 °C for 3 h in a furnace to obtain porous silica blocks. The porous silica block was silanized by immersion in a 5 wt.% silane (3-methacryloxypropyl trimethoxysilane) solution, followed by heat-treatment at 80 °C. The silanized block was immersed in a resin monomer mixture (urethane dimethacrylate (UDMA: 8 g), TEGDMA: 2 g, and initiator (benzoyl peroxide (BPO: 0.1 g)) and then subjected to heat polymerization at 80 °C for one day. Finally, the block-shaped silica-based PICN was obtained. The resultant block was cut into 10-mm-diameter, 1-mm-thick disks using a diamond wheel saw. The disk sample was polished using emery papers up to 2000 grit and then mirror-polished using diamond paste. The polished samples were used for microstructure observation, Vickers hardness, and wear tests.

### 2.2. Commercial PICN, Lithium Disilicate Glass, and Zirconia

Table 2 lists the details of the commercial PICN (ENA) and ceramics used in this study. The as-received ENA block was cut into a 1-mm-thick plate using a diamond wheel saw. The plate surfaces were polished in the same manner as mentioned above. The resultant samples were used for microstructure observation, the Vickers hardness test, and the wear test.

Commercial LDS and ZIR were cut into 1.5 mm-thick plates using a diamond wheel saw. The plates were sintered in a furnace according to the manufacturer’s instructions. The sintered plates were ground to 1 mm thickness and polished in the same manner as described above. The resultant samples were used for microstructure observation, Vickers hardness, and wear tests.

### 2.3. Microstructural Observation

Microstructures of the samples (EXP, ENA, LDS, and ZIR) were observed by scanning electron microscopy (FE-SEM, JCM-7000, JEOL Ltd., Tokyo, Japan). The observation conditions were an acceleration voltage of 5 kV and a working distance of 10 mm. Prior to the observation, each sample was coated with platinum via sputtering.

### 2.4. Human Teeth

Healthy wisdom teeth without caries were collected with the approval of the Ethics Committee of Kyushu Dental University (Approval Number 20-8, 10 September 2020). The extracted teeth were washed, sterilized, and stored in 0.5% chloramine-T aqueous solution. The stored teeth were used for experiments immediately after removal from the stored solution.

### 2.5. Vickers Hardness Test

The Vickers hardness of the as-fabricated samples was measured using a hardness tester (HMV-G21ST, Shimadzu Corporation, Kyoto, Japan) with a load of 200 g and a duration of 15 s. Ten measurements were performed for each sample (*n* = 10).

### 2.6. Wear Test

**Figure 1:**
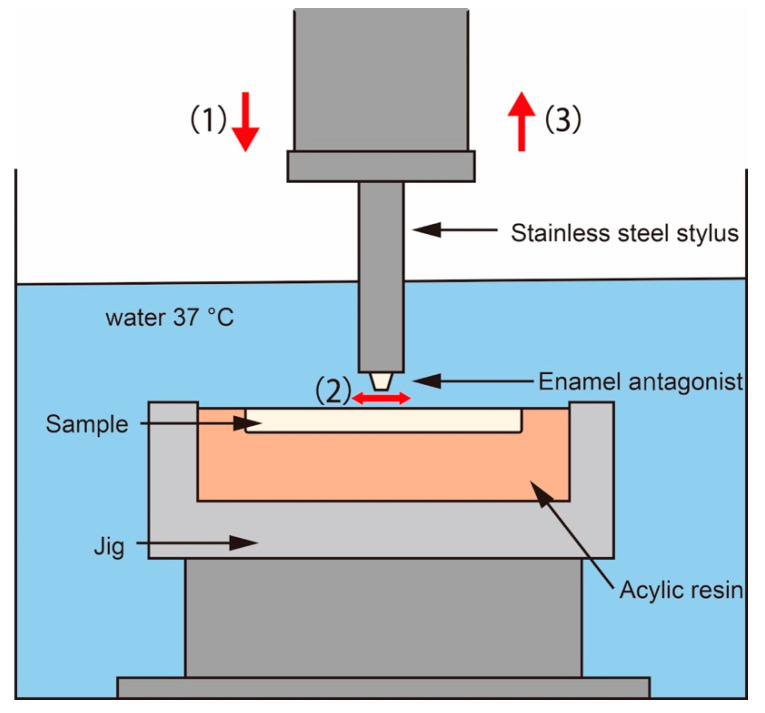
Schematic illustration of the two-body wear test in water at 37 °C. (**1**) The enamel antagonist was impacted toward the plate sample at a pressure of 0.5 MPa followed by (**2**) the sample reciprocating-sliding in the horizontal direction for 2 mm before the (**3**) stylus was retracted to the initial position. This wear process was repeated up to 50,000 cycles at a rate of 0.5 Hz.

**Figure 2:**
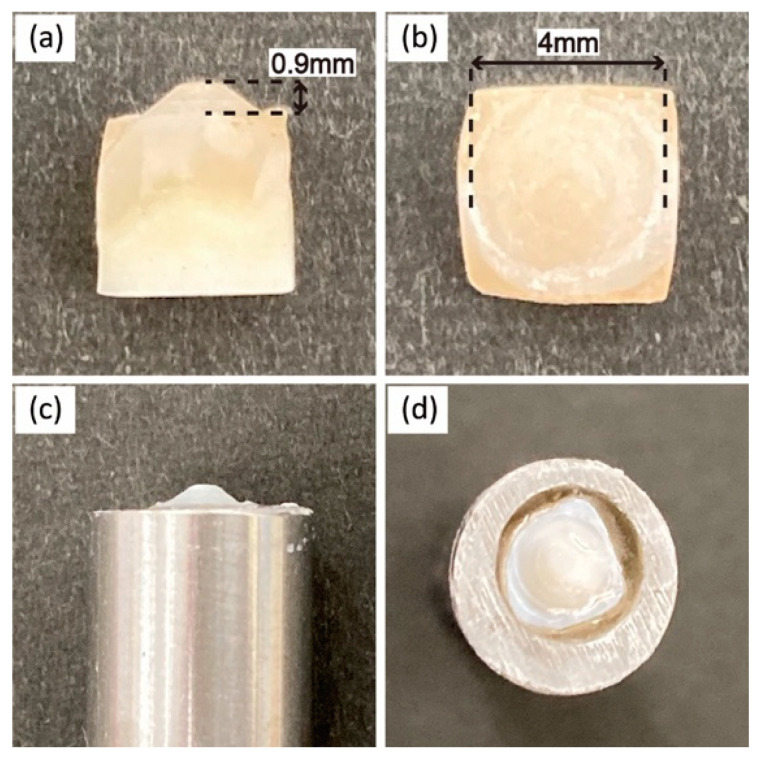
Optical images of the enamel antagonist; (**a**) side view, (**b**) top view, (**c**) side view and (**d**) top view of the antagonist mounted on the stainless-steel stylus.

**Figure 3:**
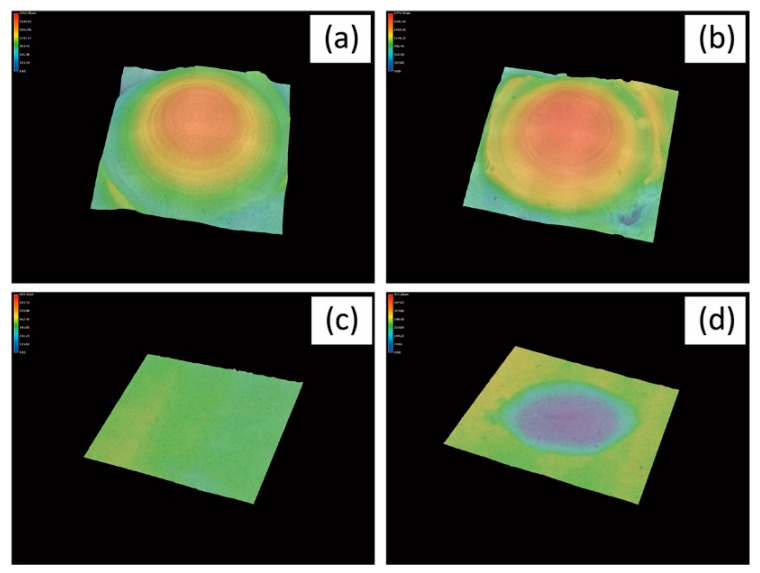
Representative 3D images for wear samples; enamel antagonists (**a**) before and (**b**) after a 50,000-cycle wear test, examined samples (**c**) before and (**d**) after a 50,000-cycle wear test.

The enamel cusps were cut out from the teeth and ground into a standardized conical shape using a diamond rotary instrument with an internal cone using the method reported by Janyavula et al. [35]. The enamel cusps were mirror-polished using diamond paste. A two-body wear test was performed using a ball-on-disc sliding wear device (K655-20, Tokyo Giken Inc., Tokyo, Japan). Figure 1 shows the wear setup. The plate sample was mounted on a jig using auto-cured acrylic resin and fixed on the device. The enamel antagonist was mounted on a stainless-steel stylus using an auto-cured acrylic resin (Figure 2) and assembled on the device. The repeating impact/slide wear was conducted in water at 37 °C for up to 50,000 cycles. The applied pressure was controlled by 0.5 MPa using a sensor installed on the device. After being subjected to different wear cycles, the sample and enamel antagonist were removed from the device, and three-dimensional measurements were taken using a digital microscope (VHX-600, Keyence Corp., Osaka, Japan). Applying a method reported in a previous study [35], the volumetric wear loss was estimated from the difference in the volume of the sample before and after the wear test using three-dimensional images (Figure 3).

### 2.7. Surface Characterization

The surface topology of each sample after the wear test was observed by SEM. Prior to observation, each sample was coated with platinum via sputtering. The surface roughness of each sample after the wear test was measured by confocal laser scanning microscopy (CLSM; VKX-100, Keyence, Osaka, Japan) with a scan area of ~300 × 300 μm^2^. Each sample was scanned five times (n = 5) at the measurement points, and the obtained surface image was analyzed using software to obtain the surface roughness, Ra.

### 2.8. Statistical Analysis

The statistical differences in the Vickers hardness and surface roughness among the groups were confirmed using software (EZR, Jichi Medical University, Saitama, Japan). Each data point was analyzed by a one-way analysis of variance (ANOVA) followed by Tukey’s post-hoc test. The significance level (*p*) was set to 0.05 in all analyses.

## 3. Results

**Figure 4:**
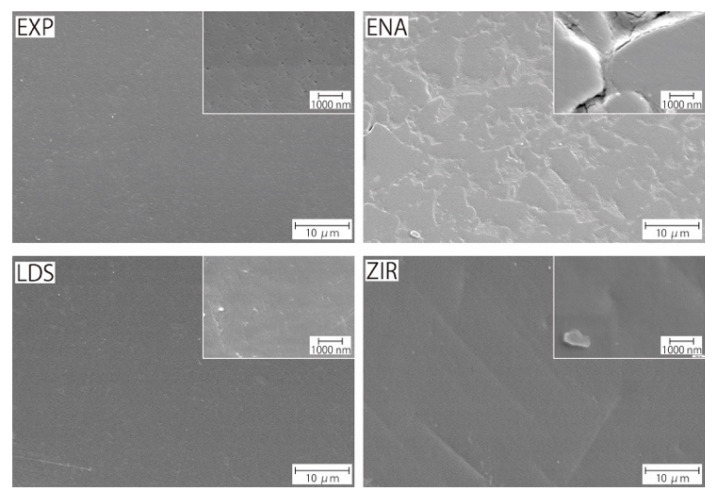
Microstructures of the as-prepared samples observed by SEM.

**Figure 5:**
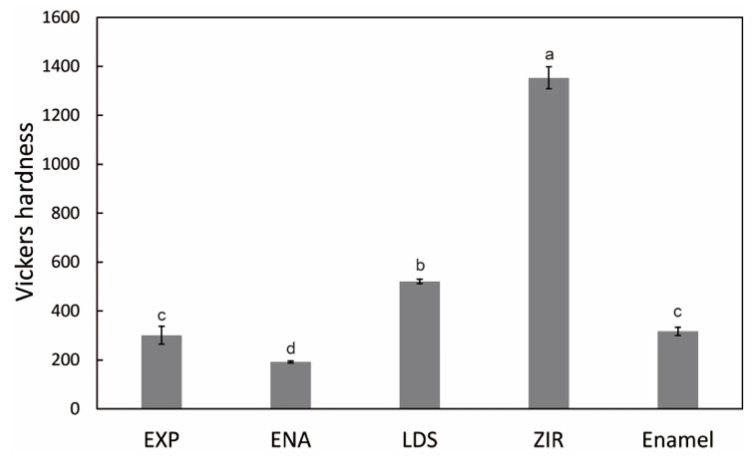
Vickers hardness of the samples. The different alphabetic letters in the figure indicate that there are significant differences between the groups (*p* < 0.05, Tukey’s test, *n* = 10).

**Figure 6:**
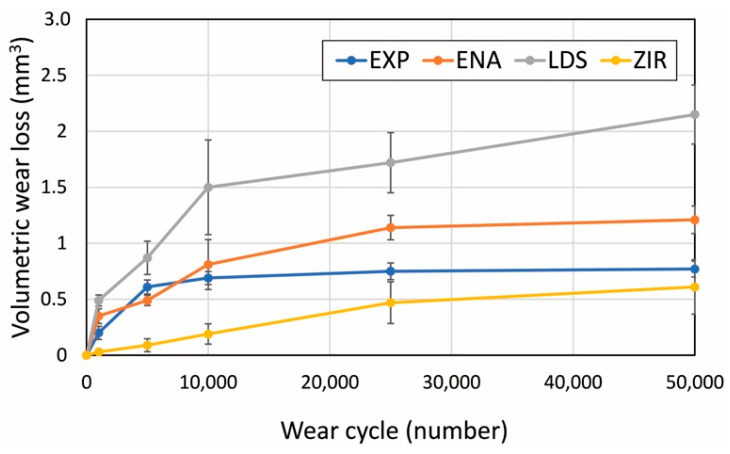
Volumetric wear loss of the enamel antagonist impacted against each sample depending on the wear cycles.

**Figure 7:**
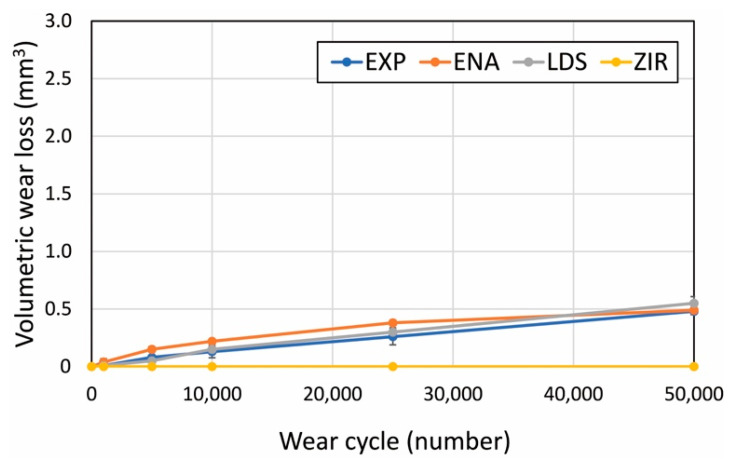
Volumetric wear loss of the EXP, ENA, LDS, and ZIR samples impacted against the enamel antagonist depending on the number of cycles.

**Figure 8:**
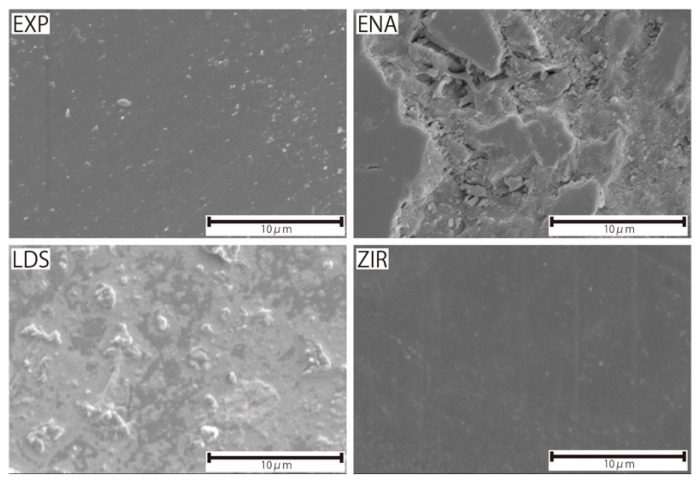
SEM images of the surfaces of the samples after a 50,000-cycle wear test.

**Figure 9:**
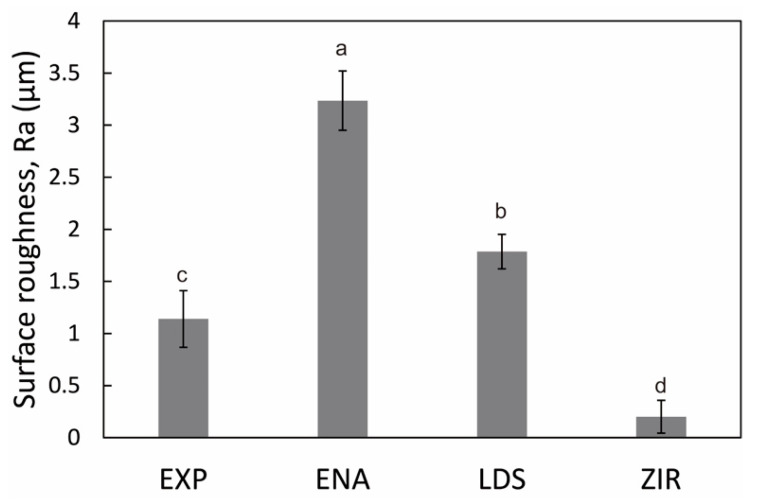
Surface roughness of the samples after a 50,000-cycle wear test measured by the CLSM. The different alphabetic letters in the figure indicate that there are significant differences between the groups (*p* < 0.05, Tukey’s test, *n* = 5).

Figure 4 presents the microstructures of the as-prepared samples before the wear test. EXP is composed of a dual-network structure of the nano-sized ceramic skeleton and infiltrated resin while ENA has the micro-sized dual-network structure. LDS and ZIR have smooth surfaces. Figure 5 shows the Vickers hardness (mean value and standard deviation) of the samples with increasing hardness in the order of ENA (192 ± 5) < EXP (301 ± 36) = enamel (317 ± 17) < LDS (521 ± 9) < ZIR (1353 ± 45). There was no significant difference between the Vickers hardness of EXP and enamel (317 ± 17). Figure 6 shows the volumetric wear loss of the enamel antagonist for each sample. In each enamel sample, the wear loss increased with an increase in the number of wear cycles. At 50,000 cycles, the volumetric wear loss (mean value and standard deviation) of the enamel is in the order of ZIR (0.61 ± 0.24 mm^3^) < EXP (0.77 ± 0.07 mm^3^) < ENA (1.21 ± 0.12 mm^3^) < LDS (2.15 ± 0.26 mm^3^). Figure 7 shows the volumetric wear loss of the samples. For EXP, ENA, and LDS, the volumetric wear losses showed similar trends that monotonically increased with an increase in the number of wear cycles. In contrast, the wear loss for ZIR was so small that it could not be measured, even after 50,000 wear cycles. Figure 8 shows SEM images of the samples after the wear test. No obvious wear damage was observed on the ZIR surface. For LDS, the crystals were exposed, such that the surface was rough. The surface of the ENA was rough due to the ceramic skeleton being exposed. The EXP had a rough surface with no exposure of the ceramic skeleton. The surface roughness due to wear was measured quantitatively (Figure 9). The surface roughness of each sample increased during the wear test, except for ZIR. The Ra values (mean value and standard deviation) were in the order of ZIR (0.20 ± 0.16 μm) < EXP (1.14 ± 0.27 μm) < LDS (1.79 ± 0.17 μm) < ENA (3.23 ± 0.29 μm).

## 4. Discussion

This study characterized the Vickers hardness and wear volumetric loss of experimental PICN (EXP), commercial PICN (ENA), lithium disilicate glass (LDS), and zirconia (ZIR) impacted against an enamel antagonist. Each sample had different microstructures that determined their mechanical properties, including the wear behavior. EXP has a dual-network structure consisting of a nanosized silica-based ceramic skeleton infiltrated by acrylic resin [34]. The mechanical properties of EXP are similar to those of human enamel in terms of Vickers hardness and to human dentin in terms of elastic modulus [34]. ENA is a CAD/CAM block used for crown restorations and is the most widely used PICN. ENA has a microstructure consisting of a micro-sized feldspar porcelain-based ceramic skeleton infiltrated with acrylic resin [36]. The chemical compositions of EXP and ENA are similar, while their microstructures are different. The ceramic skeleton of EXP is finer than that of ENA; the ceramic skeleton of the former is nano-sized, and that of the latter is micro-sized. LDS has a microstructure consisting of highly interlocking lithium disilicate crystals several micrometers in length and sub-micrometers in diameter in a silicate-based glass matrix [37,38,39]. The interlocking crystalline structure contributes to the strengthening of the glass matrix. The ZIR used in this experiment was tetragonal zirconia stabilized with 3 mol% yttria (3Y-TZP). The microstructure of 3Y-TZP is composed of high-density sintered polycrystalline structures [40]. 3Y-TZP exhibits high strength and high hardness [40].

The Vickers hardness values of ZIR and LDS are higher than that of enamel, indicating that these ceramics can potentially damage enamel antagonists. Meanwhile, the hardness of both PICNs, especially EXP, was close to that of the enamel. This suggests that EXP and ENA have a relatively low risk of damaging enamel antagonists compared with ceramics. However, it should be noted that both EXP and ENA are composed of ceramic skeletons that are harder than hydroxyapatite, including enamel. Ceramic skeletons, including EXP and ENA, may abrade enamel antagonists when they are exposed.

Wear damage on the samples is caused not only by mechanical abrasion but also by chemical degradation caused by the hydrolysis reaction in water. The PICNs (EXP and ENA) are composed of infiltrating resin phases, in addition to the ceramic skeleton. The resin phase has relatively lower physicochemical stability than the ceramic skeleton, leading to water sorption and dissolution into water. According to a previous study, deterioration of the mechanical properties due to water absorption was observed in PICN [41]. For LDS, it has been reported that the glass-matrix phase deteriorates in water via hydrolysis reactions [42]. Therefore, it is speculated that the volumetric wear losses for EXP, ENA, and LDS can be attributed to mechanochemical reactions due to abrasion and water degradation. On the other hand, the physicochemical properties of ZIR are stable and are deteriorated by hydrolysis reactions, which are unlikely to occur in the present experiment as well as in the oral environment [43].

The volumetric wear losses for the enamel antagonist against EXP and ENA were lower than those of LDS and higher than those of ZIR. In addition to the hardness of the samples, the roughness of the sample surface influences the wear progression of the enamel antagonists. Even if the initial sample surfaces are polished to make them smooth and flat, the surfaces are gradually degraded and roughened during repeated wear. As the experimental results reveal, the sample surfaces became rough after the wear test. The rough surfaces of the samples can abrade enamel antagonists. In contrast, the ZIR sample surface did not roughen even after the 50,000-cycle wear test. The smooth surface did not abrade the enamel antagonist, even though the hardness of ZIR was significantly higher than that of the enamel. This is consistent with previously reported results, suggesting that mirror-polished ZIR does not wear enamel compared to surface-roughened ZIR or glass ceramic [35]. However, it is noted that ZIR does not wear the enamel antagonist only when the surface is mirror-polished and is not coated by another material. In clinical situations, ZIR crowns are often coated with glazed porcelain. The glazed ZIR surfaces are rough and abrade the enamel antagonist by wear [44].

Overall, it was found that the PICN materials, especially the experimental PICN, wear themselves as well as enamel antagonists. However, the PICN materials do not wear enamel antagonists at the same fast rate as LDS. The wear behavior of enamel antagonists against PICN materials is therefore appropriate in clinical practice because LDS has been widely used as a crown-restorative material for over 20 years [39]. The PICN materials were also abraded by the enamel antagonists. From the viewpoint of mechanical compatibility, the wear of both crown and enamel antagonists is acceptable because healthy and unrestored natural teeth wear each other by chewing and bruxism. A small amount of wear of the crown-restorative material is necessary for maintaining appropriate occlusion. Hence, PICN materials are mechanically compatible with enamel in terms of their wear behavior. For practical crown restoration, flexural strength is one of the important mechanical properties. The flexural strength of the experimental PICN is ~150 MPa [34], which is higher than that of commercial PICN (~140 MPa) [45], while lower than those of LDS (~350 MPa) [46] and ZIR (1100 MPa) [46]. The experimental PICN would be used for single crown excepting multi-tooth restoration.

## 5. Conclusions

Within the limitations of this study, we verified that the experimental silica-based PICN has hardness and wear compatibility with human enamel. PICN is a stable material for crown restoration applications. However, the present in vitro experiment differs from the actual wear situation in an oral environment. The wear of crown and enamel antagonists caused by chewing and bruxism is complex. Many other factors such as food, pH fluctuations, temperature fluctuations, and bacteria should be considered. In the future, further clinical studies are needed to clarify the wear behavior of PICN materials for crown-restorative materials.

## Figures and Tables

**Table 1:** Reagents list used for preparing the experimental PICN.

Acronym	Material Type	Reagent (Product Name)	Purity (%)	Manufacturer
SiO_2_	Fused silica glass	Silica nanoparticles (Aerosil^®^ OX50)	≥99.8	Nippon Aerosil Co, LTD., Tokyo, Japan
HEMA	Monomer	2-Hydroxyethyl methacrylate	≥95.0	FUJIFILM Wako Pure Chemical Corp., Osaka, Japan
TEGDMA	Monomer	Triethylene glycol dimethacrylate	≥90.0	FUJIFILM Wako Pure Chemical Corp., Osaka, Japan
POE	Solvent	2-Phenoxyethanol	≥99.0	FUJIFILM Wako Pure Chemical Corp., Osaka, Japan
PrOH	Solvent	1-Propanol	≥99.5	FUJIFILM Wako Pure Chemical Corp., Osaka, Japan
BAPO	Photo-initiator	Phenylbis (2,4,6-trimethylbenzoyl) phosphine oxide	>96.0	Tokyo Chemical Industry Co., Ltd., Tokyo, Japan
γ-MPTS	3-methacryloxypropyl trimethoxysilane	Silane coupling agent	≥99.9	Shin-Etsu Chemical Co., Ltd. Tokyo, Japan
UDMA	Monomer	Urethane dimethacrylate	≥97.0	Sigma–Aldrich Co. LLC., St. Louis, MO, USA
BPO	Thermal-initiator	Benzoyl peroxide	≥97	Alfa Aesar, Haverhill, MA, USA

**Table 2:** Commercial dental PICN and ceramics.

Abbreviation	Material Type	Product Name	Manufacturer
ENA	Polymer-infiltrated ceramic network material	VITA ENAMIC	VITA Zahnfabrik, Bad Säckingen, Germany
LDS	Lithium disilicate glass	IPS e.max CAD	Ivoclar Vivadent Inc., Amherst, NY, USA
ZIR	Tetragonal zirconia polycrystal stabilized with 3 mol% yttria (3Y-TZP)	IPS e.max ZirCAD	Ivoclar Vivadent Inc., Amherst, NY, USA

## Data Availability

The data presented in this study are available on request from the corresponding author.
